# MK-7 and Its Effects on Bone Quality and Strength

**DOI:** 10.3390/nu12040965

**Published:** 2020-03-31

**Authors:** Toshiro Sato, Naoko Inaba, Takatoshi Yamashita

**Affiliations:** 1 R&D division, J-OILMILLS, Inc., Yokohama 230-0053, Japan; naoko.inaba@j-oil.com; 2 Fine Chemical Division, J-OILMILLS, Inc., Tokyo 104-0044, Japan; takatoshi.yamashita@j-oil.com

**Keywords:** vitamin K2, menaquinone-7, osteocalcin, bone metabolism, bone quality

## Abstract

Vitamin K acts as a cofactor and is required for post-translational γ-carboxylation of vitamin K-dependent proteins (VKDP). The current recommended daily intake (RDI) of vitamin K in most countries has been established based on normal coagulation requirements. Vitamin K1 and menaquinone (MK)-4 has been shown to decrease osteocalcin (OC) γ-carboxylation at RDI levels. Among the several vitamin K homologs, only MK-7 (vitamin K2) can promote γ-carboxylation of extrahepatic VKDPs, OC, and the matrix Gla protein at a nutritional dose around RDI. MK-7 has higher efficacy due to its higher bioavailability and longer half-life than other vitamin K homologs. As vitamin K1, MK-4, and MK-7 have distinct bioactivities, their RDIs should be established based on their relative activities. MK-7 increases bone mineral density and promotes bone quality and strength. Collagen production, and thus, bone quality may be affected by MK-7 or MK-4 converted from MK-7. In this review, we comprehensively discuss the various properties of MK-7.

## 1. Introduction

Vitamin K acts as a cofactor to γ-glutamyl carboxylase (GGCX), an enzyme that catalyzes glutamic acid residues of specific proteins to γ-carboxyglutamic acid (Gla) to form Gla-containing proteins. These proteins, also called vitamin K-dependent proteins (VKDPs) [1,2,3,4,5,6,7,8,9,10,11], are listed in Table 1. When intake of vitamin K is insufficient, VKDPs are not fully activated and fail to execute their specific functions.

Numerous blood coagulation factors, including coagulation factors II (prothrombin), VII, IX, and X, and anti-coagulation factors, such as proteins C, S, and Z, are well-known examples of VKDPs and are synthesized in the liver [1,2]. Thus, vitamin K is an indispensable nutrient for normal blood coagulation, and its deficiency rarely occurs in adults.

Functions of extrahepatic VKDPs have been extensively studied. Several VKDPs play important roles in maintaining bone metabolism and inhibiting ectopic calcification, leading to bone and cardiovascular health improvement [12]. For example, osteocalcin (OC) is synthesized by osteoblasts, the matrix Gla protein (MGP) is synthesized in vascular smooth muscle cells, cartilage, and bones, and the Gla-rich protein (GRP) is expressed in the cartilage and bones. Growth arrest-specific protein 6 (Gas6) expressed in the brain is involved in cell proliferation. On the other hand, the functions of certain VKDPs, that is, proline-rich Gla protein 1 (PRGP1), PRGP2, transglutaminase 3 (TGM3), and TGM4 are still not well-known. The VKDP GGCX is also expressed in almost all tissues. and there might be other VKDPs that are yet to be discovered.

In addition to activating various VKDPs, vitamin K acts as an antioxidant [13] and vitamin K2 or menaquinone-4 (MK-4) acts as a ligand of the steroid and xenobiotic receptor/pregnenolone X receptor (SXR/PXR) [14,15]. Thus, vitamin K is expected to have various health benefits [13], preventing or alleviating cardiovascular disease, bone fracture, diabetes mellitus, cancer, liver disease, chronic kidney disease, immune disorder, neurological disease, and obesity.

Recently, vitamin K2 (MK-7) has been found to be highly effective in activating extrahepatic VKDPs at nutritional doses. In this study, we review the properties of MK-7.

## 2. Sources of Vitamin K

The two naturally occurring forms of vitamin K are vitamin K1 (phylloquinone) and vitamin K2 (menaquinone (MKs or MK-n)). Vitamin K1 occurs in various green vegetables and plant oils and is the primary dietary source of vitamin K [16]. Vitamin K1 is present in the chloroplast membrane of leafy green vegetables. Vitamin K2 has a variable side chain length of four to 15 isoprene units and is referred to as MK-n, where n denotes the number of isoprenoid units. A small amount of MK-4 is found in animal products, such as eggs, meat, and liver. MK-4 in animal foods results from the conversion of vitamin K1 in plant feed or menadione (a synthetic analog of vitamin K, containing only the 2-methyl-1,4-naphthoquinone ring structure), provided to animals as a feed additive [17,18]. The long chain MKs, such as MK-7–MK-9, are found in fermented foods. These MKs are also bacterial products found in fermented foods [19,20]. A Japanese traditional food, natto, is a unique soybean product fermented with a specific kind of *Bacillus subtilis* and contains MK-7 at a very high concentration [19,20].

Bacteria present in the colon produce a substantial quantity of long chain MKs [21,22]. The extent to which intestinal bacteria-derived MKs contribute to the daily requirement of vitamin K has been a matter of debate [23,24]. However, it is thought that MKs derived from intestinal bacteria are difficult to be absorbed in the distal digestive tract, and some experiments have shown that MKs derived from intestinal flora alone are insufficient [25,26].

**Figure 1:**
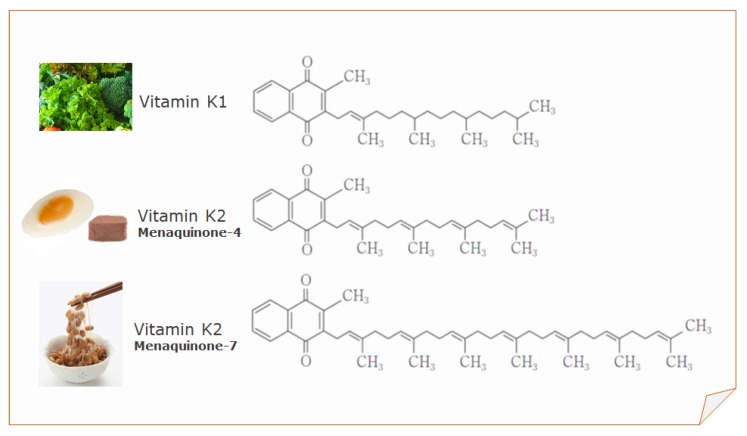
Structure of vitamin K1, menaquinone-4 (MK-4), and menaquinone-7 (MK-7).

Vitamin K1, MK-4, and MK-7 are currently used for fortification purposes by the food industry and as nutritional supplements (Figure 1).

## 3. Daily Requirement of Vitamin K

Currently, the recommended daily intake (RDI) or adequate intake (AI) of vitamin K is based on the maintenance of normal blood coagulation [27,28]. The National Academy of Medicine in the US has set the AI of vitamin K1 at 120 µg/day for adult men and 90 µg/day for adult women [29]. The World Health Organization and the Food and Agriculture Organization have set the recommended dosages for vitamin K1 at 65 µg/day for men and 55 µg/day for women, on the basis of 1 µg/day/kg body weight [30]. The European Commission has set the recommended daily allowance (RDA) for vitamin K at 75 µg/day [31]. Japan has set the AI of vitamin K at 75 µg/day for adult men and 65 µg/day for adult women by the Ministry of Health, Labor, and Welfare in 2010 [32], with both values being over 1 µg/day/kg body weight.

**Figure 2:**
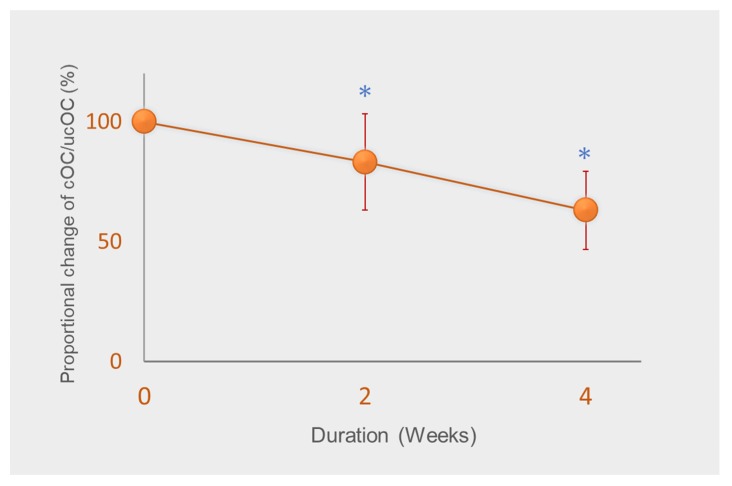
Change in the ratio of carboxylated osteocalcin (cOC) to undercarboxylated osteocalcin (ucOC) from baseline. Subjects were administered 72 µg vitamin K/day (around adequate intake of vitamin K) for four weeks. Data are expressed as mean ± standard deviation of 14–15 subjects. * Significantly different from baseline, *p* < 0.001. Adapted from [35].

However, studies have suggested that relatively higher vitamin K intake is required for bone and vascular health [33]. As vitamin K accumulates mostly in the liver and is used for coagulation, a greater quantity is thought to be required for extrahepatic tissues [34]. In a previous study, we demonstrated the AI for vitamin K for Japanese adult women set in 2010, that is, 65 µg/day, is insufficient for the γ-carboxylation of osteocalcin (OC) [35]. Vitamin K intake from meals of all subjects was strictly controlled to an average of 72 μg/day, mainly with vitamin K1 and small amounts of MK-4 throughout the study. We analyzed serum carboxylated OC (cOC) and undercarboxylated OC (ucOC), and determined the ratio of cOC/ucOC, a sensitive marker of vitamin K status in bone. The ratio of cOC/ucOC significantly decreased after two weeks and decreased further by about 40% from baseline (Figure 2) [35]. Furthermore, cOC and ucOC concentrations continued to exhibit a tendency to decrease. Because daily intake of 72 μg/day corresponds to 1.3 µg/day/kg body weight, the results demonstrated that the current RDIs of vitamin K set by many countries are insufficient for the γ-carboxylation of OC. It should be noted that fabricated diets, such as enteral feeding products and multi-nutrient diets for hospitals prepared based on RDIs may result in vitamin K deficiency for bone metabolism, affecting bone health and increasing bone fracture risk.

In our study [35], an additional intake of 50 μg/day of MK-7 protected the OC carboxylation rates or cOC/ucOC. In 2015, the Japanese daily AI for vitamin K for adults was increased almost two-fold to 150 µg/day for both adult men and women. However, it is still unknown whether this level is sufficient for normal bone metabolism, as vitamin K1 from vegetables is poorly absorbed [20].

As vitamin K1, MK-4, and MK-7 have different intestinal absorption rates and blood half-lives in humans, their physiological activities are also considered to differ [36,37]. Thus, the RDIs for vitamin K homologs should be established based on their relative activities.

When higher doses over the RDA were used, no hypercoagulable state was observed [38]. The adverse effects of MK-7 has not been reported by prolonged supplementation of MK-7 for 3 years [39,40]. We found that an intake of 600 μg/day of MK-7 for one month did not affect biochemical parameters in serum and urine in healthy subjects. Safety of MK-7 has been reviewed by Marles et al. [28], and its use in food for fortification purposes has been approved in many countries.

## 4. MK-7 and Bone Quality

**Figure 3:**
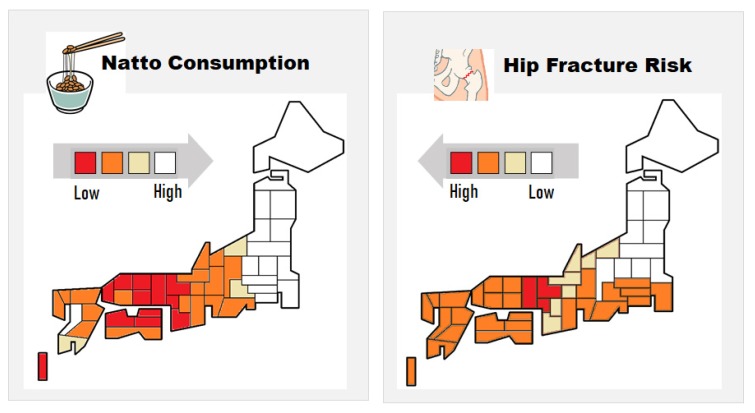
Correlation between the regional relative incidence of hip fractures and natto consumption in Japanese women. Adapted from [41].

Natto, fermented soybean specifically produced by *B. subtilis*, is a traditional Japanese food high in MK-7 (200–400 µg per serving of 30–45 g). Due to its distinctive strong smell, stickiness, and texture, natto consumption varies markedly depending on the region. Regional studies have shown that natto consumption reduces incidences of hip fractures in women in Japan (Figure 3) [41,42]. Recently, a large prospective cohort study revealed that natto intake is inversely correlated with fracture risk [43]. In this study, frequency of intake of other soybean products had no association with fracture risk. The major difference between natto and other soybean products is that the former is prepared by fermentation using *B. subtilis* and contains a high amount of MK-7. Thus, the higher MK-7 levels due to natto consumption may have contributed to the relatively lower fracture risk [41,42,43]. Natto has been eaten for centuries in Japan, and no particularly problematic side effects have been reported. However, patients taking vitamin K antagonists (VKAs), such as warfarin, should refrain from taking natto, which may affect the stability of VKAs.

**Figure 4:**
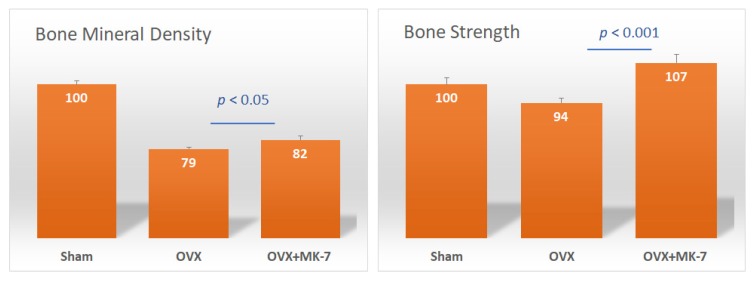
Effect of menaquinone-7 (MK-7) on bone mineral density (BMD) and bone strength of the femurs of ovariectomized rats. Sham: sham-operated group; OVX: ovariectomized rat control group; OVX + MK-7: ovariectomized rats that were fed MK-7. Data are expressed as relative values with the sham group taken as 100%. Adapted from [45].

The association of frequent natto intake with a reduced risk of osteoporotic fractures was shown to be independent of bone mineral density (BMD), suggesting that natto is beneficial for bone quality [43]. A study reported that administration of MK-7 for six weeks did not have any effects on bone strength and bone mineral density in ovariectomized rats [44]. However, another study showed that intake of MK-7 for five months prevented BMD loss to a certain extent, but significantly improved bone strength in rats (Figure 4) [45]. Therefore, the primary benefits of MK-7 are maintaining and improving bone quality, thus improving bone strength rather than increasing BMD. A clinical study demonstrated that postmenopausal women treated with a pharmacological dose of MK-4 (45 mg/day) for three years showed no effects on BMD, but bone quality indices of the femur increased [46]. In addition, MK-7 (180 µg/day) was demonstrated to inhibit bone loss and helped maintain high bone strength in healthy postmenopausal women [39].

In addition to OC carboxylation, which modulates the deposition of calcium in bone, MK-4 increases collagen accumulation [47]. We also confirmed that MK-7 increased collagen production using osteoblasts [48]. Collagen is essential to bone flexibility and elasticity, and occupies more than half the volume of bones. It is responsible for matrix production, the material on which calcium and other minerals accumulate. Therefore, along with bone minerals, collagen accumulation is critical for high-quality bone formation.

Other than OC, many VKDPs, such as MGP, protein S [49], and periostin are produced in the bone matrix, suggesting a complex involvement of vitamin K and VKDPs in bones.

## 5. Advantages of MK-7

Osteocalcin has been used as a biomarker for bone metabolism. Vitamin K deficiency leads to an increase in serum ucOC, and a high serum ucOC level has been associated with hip fractures [50,51], and has been recognized as an independent risk factor for fractures. Since 2007, serum ucOC has been used as a diagnostic marker to evaluate vitamin K deficiency in bones in Japan. A smaller dose of MK-7 can γ-carboxylate OC compared to doses of K1 or MK-4. A supplemental intake of 250–1000 µg/day of vitamin K1 activates OC [52], which is higher than the current RDIs of vitamin K in most countries. A markedly higher dose of MK-4 (600–1500 µg/day) is required to activate OC [53,54], as it has been shown to have a very short half-life in humans [37] and it is poorly absorbed [55]. Nutritional doses of MK-4, such as the consecutive intake of 60 µg/day or a single intake of 420 μg, have shown to be ineffective [55]. In contrast, MK-7 at doses around the current RDI (90–180 µg/day) promoted OC carboxylation [35,38,56]. A study demonstrated that MK-7 derived from natto has a very long half-life in the serum and induces more complete carboxylation of OC compared to vitamin K1 in humans [57].

As all vitamin K homologs are converted to MK-4 in tissues, MK-4 is considered to perform other specific functions other than γ-carboxylation of VKDPs [58,59]. However, in our previous study [60], the intake of a nutritional dose of MK-4 did not lead to an increase in MK-4 levels in the extrahepatic organs of rats, while that of MK-7 led to a significant increase in MK-4 in organs such as the femur, brain, and testis. This implies that to achieve MK-4-specific physiological effects, it might be better to intake MK-7 as a MK-4 precursor than MK-4 itself.

In addition to γ-carboxylation of VKDPs and the SXR-receptor ligand by conversion of MK-7 to MK-4, MK-7, the precursor of MK-4, directly activates bone formation by osteoblasts [61] and suppresses bone resorption [62]. It has also been shown that MK-7 stimulates osteoblastogenesis and suppresses osteoclastogenesis by inhibiting the activation of NF-κB [63].

## 6. Conclusions

Among vitamin K homologs, MK-7 has been shown to have the highest bioavailability and the most significant effect on OC carboxylation in humans. Vitamin K1 and MK-4 at their current RDIs are not sufficient for activation of OC. On the other hand, it is expected that MK-7 may promote bone health.

## Figures and Tables

**Table 1:** Vitamin K-dependent proteins.

Protein	Function	Ref
Factors II (Prothrombin), VII, IX, X	Procoagulants	[1,2]
Proteins C, S, Z	Anticoagulants	[1,2]
Osteocalcin	Regulator of mineral deposition	[3]
Matrix γ-carboxyglutamic acid protein	Inhibition of ectopic calcification	[4]
γ-carboxyglutamic acid-rich protein	Inhibition of ectopic calcification, anti-inflammatory	[5]
Periostin	Inhibition of ectopic calcification, tissue regeneration	[6]
Growth arrest-specific protein 6	Cell proliferation	[7,8]
Proline-rich γ-carboxy glutamyl proteins 1 and 2	Not well-known	[9,10]
γ-glutamyl carboxylase	γ-glutamyl carboxylation of vitamin K-dependent proteins	[11]
